# Antireflection of optical anisotropic dielectric metasurfaces

**DOI:** 10.1038/s41598-023-28619-8

**Published:** 2023-01-30

**Authors:** Yu-Hsuan Liao, Wei-Lun Hsu, Chen-Yi Yu, Chih-Ming Wang

**Affiliations:** Department of Optics and Photonics, National Central University, Taoyuan, 32001 Taiwan

**Keywords:** Metamaterials, Nanophotonics and plasmonics

## Abstract

We propose a hetero-nano-fin structure to further improve the efficiency of Pancharatnam–Berry phase metasurfaces. Two hetero-nano-fin types, MgF_2_/GaN and MgF_2_/Nb_2_O_5_, were investigated. The overall polarization conversion efficiency (PCE) improved from 52.7 to 54% for the MgF_2_/GaN nano-fin compared with the bare GaN nano-fin. The overall PCE of the Nb_2_O_5_ nano-fin was 1.7 times higher than that of the GaN nano-fin. The overall PCE improved from 92.4% up to 96% after the application of MgF_2_ antireflection. Moreover, the antireflection improves efficiency by an average of 4.3% in wavelengths from 450 to 700 nm. Although the increment of energy seems minimal, antireflection is crucial for a metasurface, not only enhancing efficiency but also reducing background signal of a meta-device.

## Introduction

Plasmonic metasurfaces, consisting of two-dimensional metallic meta-atom arrays, are advantageous because of their properties such as ultrathin thicknesses, ease of fabrication, versatile functionality, field confinement beyond the diffraction limit, and superior nonlinearity^1–6^. Although a plasmonic metasurface has an impressive light modulation ability, its efficiency is limited as it suffers from Ohmic heat loss during resonance. Huygens meta-atoms, which are meta-atoms in which resonances of the electric dipole and magnetic dipole coincide, can break the scattering symmetry and improve the transmission efficiency^7,8^. Although zero back-scattering can be achieved under the first Kerker condition^9^, the transmission efficiency of a plasmonic metasurface is limited at a visible frequency.

A dielectric metasurface almost effortlessly avoids Ohmic heat loss and has therefore rapidly attracted extensive research attention. In addition to resonance-based light modulation, the dielectric metasurface is generally capable of two typical light modulation methods: propagation phase^10–14^ and geometric phase^15–21^. The propagation phase involves phase accumulation during light propagation. For a fixed propagation path, the effective index of the metasurface determines the optical path difference (OPD), such as controlling the filling ratio of the material. The geometric phase is also termed the Pancharatnam–Berry (PB) phase. Light carries an additional phase when it passes through an optical anisotropic structure/material. Spatially varying geometric orientation of the optical anisotropic structure can control phase distribution. Moreover, the original polarization state of light changes to the orthogonal one (e.g., left-hand circular polarization to right-hand circular polarization) and carries geometric phase modulation. Therefore, the PB-phase metasurface also acts as a half-waveplate for polarization conversion. However, the half-waveplate condition does not coincide with the antireflection condition. Moreover, materials with a high refractive index are suitable as dielectric metasurfaces; however, they usually lead to impedance mismatch at the interface. Consequently, the PB-phase metasurface suffers from severe Fresnel reflection loss.

A traditional antireflective thin-film consists of single or multiple homogeneous layers and has refractive indices and thicknesses suitable for reducing the Fresnel reflection at the interface. Sub-wavelength structures, such as gratings^22^, pillars^23,24^, pyramids^25,26^, moth-eyes^27–29^, and nanopores^30^, may also be used for antireflection. These structures generate a gradient effective refractive index to reduce the refractive index contrast between air and the medium through which light is entering. Nowadays, sub-wavelength structure-based antireflection has been widely used for photovoltaic solar cells^31–36^.

Macroscopically, a dielectric PB-phase metasurface is an optically anisotropic medium that is similar to a birefringent structure. In 2001, Mohammed et al. applied an antireflection coating on smooth surfaces by exploiting the anisotropic nano-topology of a liquid–crystal polymer film^37^. In 2018, Zhu used three laminating layers as a broadband antireflection coating for a birefringent sapphire waveplate^38^. The aforementioned studies investigated reflection from a homogeneous and anisotropic layer. However, microscopically, as the dimension of meta-atoms is close to the incident light wavelength, the metasurface should be considered an inhomogeneous, but not a homogeneous, anisotropic medium.

In this work, we investigated the antireflection layer for the PB-phase metasurface. First, we discussed whether the traditional antireflection layer is suitable for the dielectric PB-phase metasurface. Then, an antireflection structure was proposed to enhance the efficiency of the metasurface. We then numerically investigated the overall polarization conversion efficiency (PCE) and optical properties of both gallium nitride (GaN) and niobium oxide (Nb_2_O_5_) nano-fins with an antireflection structure. To obtain the highest PCE, the thickness of the nano-fin was corresponded to a half-waveplate. Magnesium fluoride (MgF_2_), a common low-index material for antireflection, was chosen as the antireflection layer. Here, we simulated the overall PCE enhancement and optical properties with three different arrangements of the MgF_2_ layer. Based on the calculated results, an antireflection structure for the dielectric PB-phase metasurface was proposed. Thus, antireflection for the metasurface is crucial for enhancing the efficiency as well as reducing the background signal of a meta-device.

## Results and discussions

**Figure 1:**
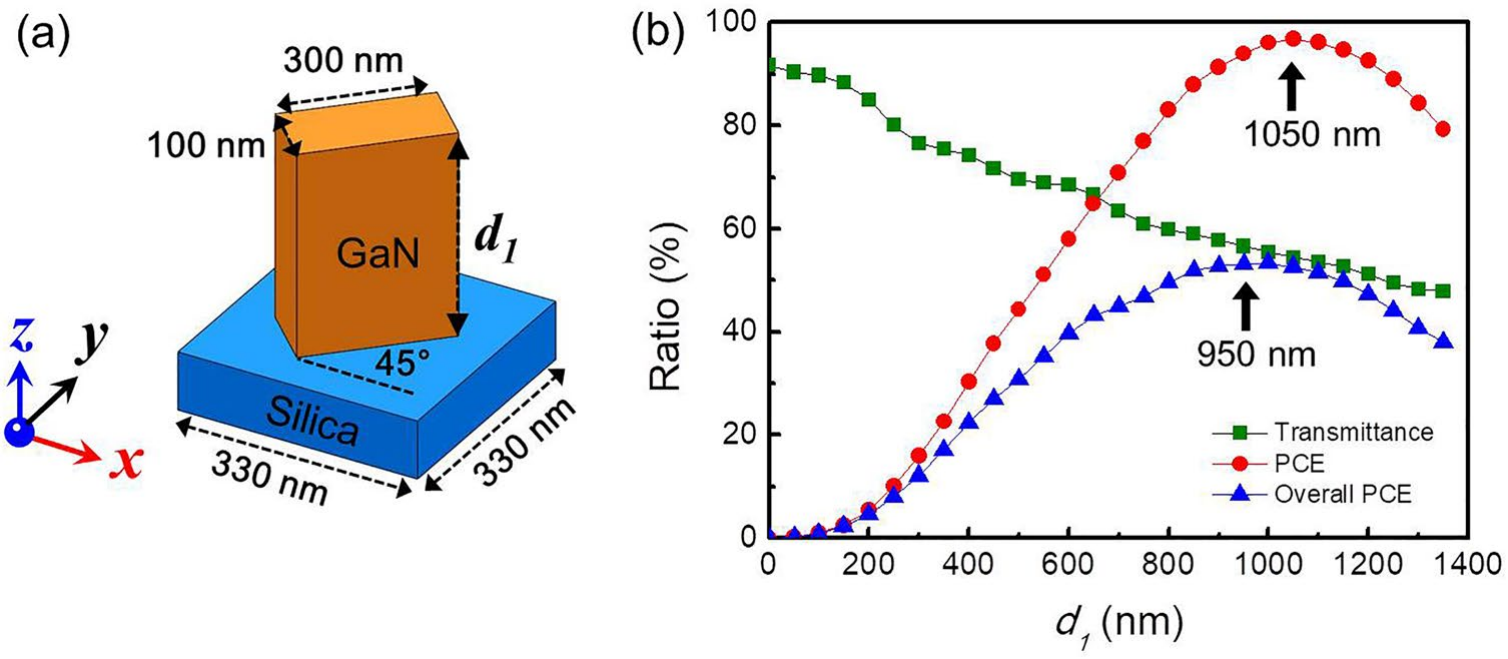
Geometric diagram and optical property of GaN nano-fin. (**a**) Geometric diagram of a GaN nano-fin on a silica substrate, where *d*_*1*_ is the thickness of the nano-fin. (**b**) Transmittance, PCE, and overall PCE versus the thickness of the nano-fin under light illumination of 633 nm at normal incidence along the z-direction from bottom to up. The polarized light is along the x-direction. The light is x-polarized, with λ = 633 nm. The light impinges perpendicularly onto the structure from bottom along the positive z-direction.

As shown in Fig. 1a, the basic building block is a GaN nano-fin on a silica substrate. The nano-fin had its basic dimensions fixed (length: 300 nm, width: 100 nm, and period: 330 nm), while its thickness (*d*_*1*_) was varied for the analysis of corresponding optical properties. All simulations were performed using the finite-difference time domain method, which is used to simulate the PCE and transmittance. In this work, incident light was assumed to be normally incident at a wavelength of 633 nm. The polarization state was x-linear polarization (XLP) propagating along the z-direction from bottom to up, as shown in Fig. 1a. The inclined angle between the x-axis and the long axis of the nano-fin was set at 45°. Under this assumption, the PCE and transmittance were identical to a circular polarized light normally incident on a nano-fin at an arbitrary rotation angle. The XLP incident light was denoted by *E*_*x-in*_. After passing through the nano-fin, the polarization state varied because of the anisotropy of the nano-fin. Therefore, the output component of the *E*-field (denoted by *E*_*out*_) consisted of *E*_*x-out*_ and *E*_*y-out*_, which represented *E*_*x*_ and *E*_*y*_ components at the output plane, respectively. The PCE can be calculated as follows:

1 $$ \frac{{|E_{y - out} |^{2} }}{{|E_{out} |^{2} }} \times 100\% = \frac{{|E_{y - out} |^{2} }}{{|E_{x - out} |^{2} + |E_{y - out} |^{2} }} \times 100\% $$

For PB-phase metasurfaces, the PCE is occasionally used to evaluate the efficiency. However, emphasis is mostly on the overall efficiency, which is the ratio of the signal light intensity to the incident light intensity. Therefore, transmittance and overall PCE must be determined.

The transmittance is defined as follows:

2 $$ \frac{{{|}E_{out} |^{2} }}{{|E_{x - in} |^{2} }} \times 100\% = \frac{{|E_{x - out} |^{2} + |E_{y - out} |^{2} }}{{|E_{x - in} |^{2} }} \times 100\% $$

Finally, the overall PCE can be defined as PCE multiplied by transmittance, as given below:

3 $$ \frac{{|E_{y - out} |^{2} }}{{|E_{x - in} |^{2} }} \times 100\% $$

The overall PCE is more appropriate for describing the overall efficiency of a PB-phase unit cell, which considers both transmittance and PCE. Figure 1b depicts the transmittance, PCE, and overall PCE as a function of the thickness (*d*_*1*_) of the GaN nano-fin. The square, circle, and triangle represent the transmittance, PCE, and overall PCE, respectively. The peak of the PCE is at *d*_*1*_ = 1050 nm, corresponding to the half-waveplate condition for polarization conversion. However, due to the yellow-band absorption of GaN, caused by Ga vacancies or their complexes^39,40^, GaN is lossy at *λ* = 633 nm and the corresponding refractive index is n + ik = 2.29 + 0.061i^41^. Therefore, transmittance decreased with an increase in thickness. After considering the contribution of transmittance, the highest overall PCE was believed to appear at *d*_*1*_ = 950 nm.

**Figure 2:**
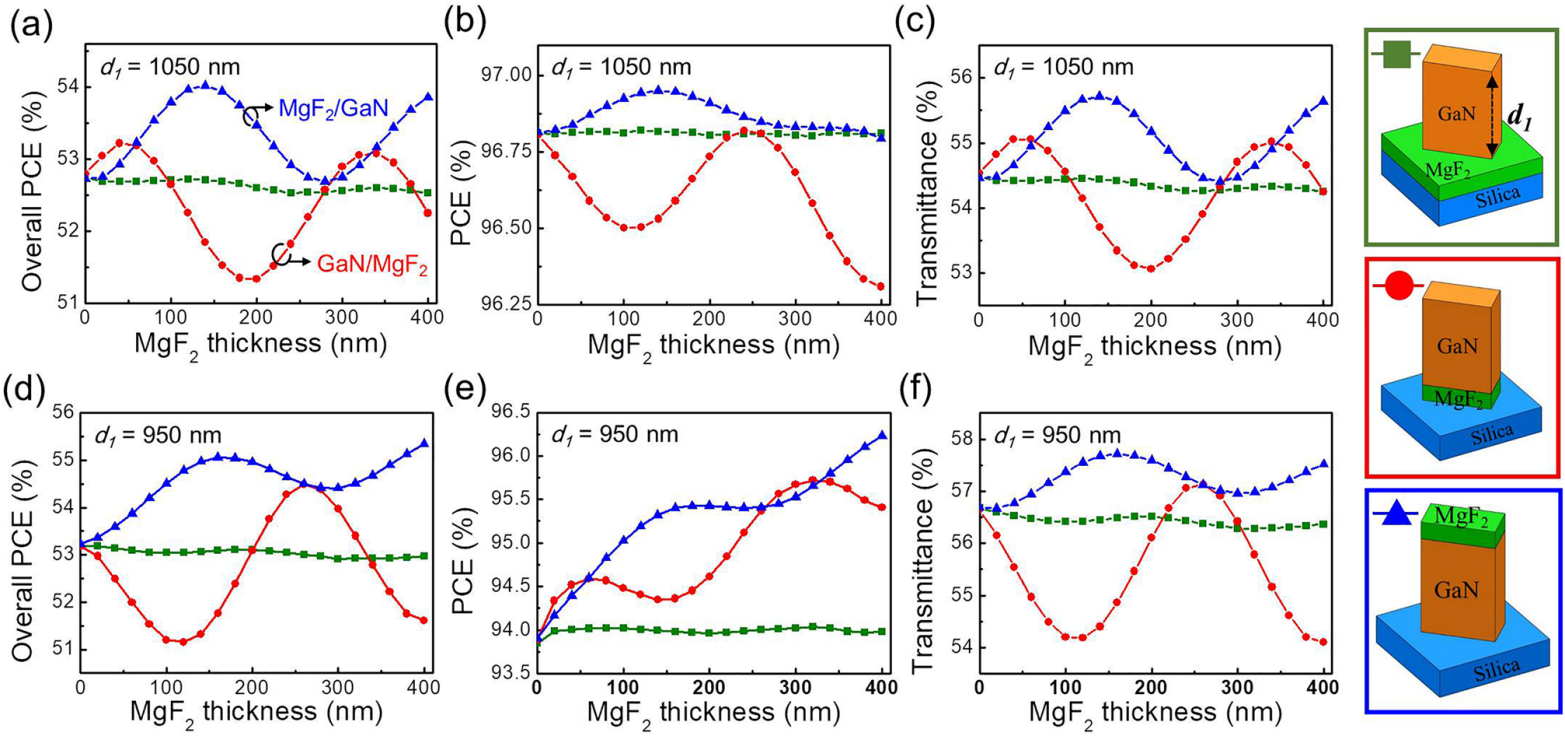
Optical property of GaN nano-fin consisted with MgF_2_ layer. Simulation results of (**a**) overall PCE, (**b**) PCE, and (**c**) transmittance versus MgF_2_ thickness with *d*_*1*_ = 1050 nm. Simulation results of (**d**) overall PCE, (**e**) PCE, and (**f**) transmittance of versus MgF_2_ thickness with *d*_*1*_ = 950 nm. The green square denotes a flat MgF_2_ film deposited under a GaN nano-fin, the red circle denotes a GaN nano-fin located on the top of the MgF_2_ nano-structure, and the blue triangle denotes a MgF_2_ nano-structure stacked on the top of a GaN nano-fin.

Here, we considered three different cases of the MgF_2_-based antireflection structure for reducing the reflection loss of GaN nano-fins. The first was the GaN nano-fin deposited on a flat MgF_2_ film over a silica substrate, which is the most intuitive antireflection structure (denoted by the green square in Fig. 2. The second (denoted by the red circle) was a GaN nano-fin stacked on a MaF_2_ nano-fin, which was fabricated through standard lithography and reactive-ion etching. This is a hetero-nano-fin. For convenience, the second nano-fin is called the GaN/MgF_2_ nano-fin. Moreover, the MgF_2_ nano-structure had the same geometric parameters as the nano-fin in the x and y directions. Finally, the third nano-fin (denoted by the blue triangle) was a MgF_2_ nano-fin stacked on the GaN nano-fin and was called a MgF_2_/GaN nano-fin. Same as the previous case, the geometric parameters of MgF_2_ and GaN nano-fins were identical in the x and y directions. The simulation results of the overall PCE, PCE, and transmittance as a function of MgF_2_ thickness are displayed in Fig. 2a–c, respectively. For *d*_*1*_ = 1050 nm, the flat MgF_2_ film (green square) barely contributed to the PCE, overall PCE, and transmittance even with an increase in the thickness. Therefore, this case was used as a reference. For the GaN/MgF_2_ nano-fin (red circle), a noticeable oscillation of overall PCE was observed with an increase in MgF_2_ thickness. When MgF_2_ thickness increased, the PCE curve oscillated and became lower, which was because the anisotropic OPD of nano-fin is away from the optimal optimized thickness of the half-waveplate (as mentioned in Fig. 1b). At the same time, the GaN/MgF_2_ nano-fin positively contributed to transmittance. Although a 0.5% increase in transmittance was observed for MgF_2_ = 60 nm, the transmittance was suppressed for most thicknesses. Notably, the overall PCE was significantly improved for the MgF_2_/GaN nano-fin. The overall PCE increased from 52.7 to 54% when MgF_2_ thickness increased from 0 to 140 nm. Although the overall PCE oscillated with varying MgF_2_ thickness, the overall PCE constantly improved, compared with a bare GaN nano-fin. Because the GaN nano-fin with a thickness of 1050 nm already had the highest PCE, only a tiny increment was observed in the PCE after the addition of the MgF_2_ nano-structure. Therefore, improvement in the overall PCE is believed to be mainly contributed by transmittance. Figure 2c shows a good agreement that transmittance improves from 54.4 to 55.7%. Figure 2d–f depict the overall PCE, PCE, and transmittance for *d*_*1*_ = 950 nm, which is the optimal thickness of the bare GaN nano-fin for the overall PCE. Compared with *d*_*1*_ = 1050 nm, both GaN/MgF_2_ and MgF_2_/GaN nano-fins showed an improved overall PCE for *d*_*1*_ = 950 nm, as shown in Fig. 2d. Both PCE and transmittance exhibited significant improvement. The MgF_2_ nano-structure simultaneously played a role in polarization conversion and antireflection. The increasing thickness allowed the anisotropic OPD to match the optimal half-waveplate condition. Therefore, the PCE for both GaN/MgF_2_ and MgF_2_/GaN nano-fins improved with an increase in MgF_2_ thickness. For transmittance, behaviors of GaN/MgF_2_ and MgF_2_/GaN differed with increasing MgF_2_ thickness. Although GaN/MgF_2_ enhanced transmittance to 1.3% at a MgF_2_ thickness of 260 nm, the transmittance was generally reduced for other thicknesses of MgF_2_. By contrast, the transmittance of MgF_2_/GaN was constantly improved regardless of the variation in MgF_2_ thickness. The flat MgF_2_ layer made no contribution to overall PCE for both *d*_*1*_ = 1050 or 950 nm. This means that the hetero-structure is necessary for antireflection.

For a homogeneous medium, the Goos–Hanchen phase is π when the incident angle is less than the total reflection angle^42^. In our case, an optical anisotropic nano-fin was considered. The reflected phase is no more exactly equal to π owing to anisotropy, depolarization, and scattering. Here, a simple optical isolator was used for comparison. Right-circularly polarized (RCP) light normally impinges on an optical homogeneous and anisotropy film, that is, a half-waveplate. After traveling to the top of the film–air interface, part of the light reflects and back travels to the input plane. An antireflection structure allows incident and reflection beams to deconstructively interfere. The Jones matrix of the input and reflected light can be represented as follows:

4 $$ E_{out} = {\text{R}}\left( { - \theta } \right)\left( {\begin{array}{*{20}c} {e^{{ - i\frac{\Gamma }{2}}} } & 0 \\ 0 & {e^{{i\frac{\Gamma }{2}}} } \\ \end{array} } \right){\text{R}}\left( \theta \right)\left( {\begin{array}{*{20}c} 1 & 0 \\ 0 & { - 1} \\ \end{array} } \right){\text{R}}\left( { - \theta } \right)\left( {\begin{array}{*{20}c} {e^{{ - i\frac{\Gamma }{2}}} } & 0 \\ 0 & {e^{{i\frac{\Gamma }{2}}} } \\ \end{array} } \right){\text{R}}\left( \theta \right)\frac{1}{\sqrt 2 }\left( {\begin{array}{*{20}c} 1 \\ { - i} \\ \end{array} } \right) $$

where *θ* is the phase retardation and R is the rotation matrix:

5 $$ {\text{R}}\left( { - \theta } \right) = \left( {\begin{array}{*{20}c} {\left. {{\text{cos}}(\theta } \right)} & {\left. {{\text{sin}}(\theta } \right)} \\ {\left. { - {\text{sin}}(\theta } \right)} & {\left. {{\text{cos}}(\theta } \right)} \\ \end{array} } \right) $$

As the RCP travels at the quarter-waveplate position, the polarization state converts to XLP. At the top of the film–air interface, polarization states of the incident and reflected light are left-circularly polarized (LCR) and RCP, respectively. When light back travels to the quarter-waveplate position, the polarization state converts to y-linear polarization. Finally, at the input plane, the back-reflected light is LCP, which is orthogonal to the input light. Thus, the vector product of the input and reflected light is zero. Therefore, the input and reflected light cannot interfere, and thus, a conventional thin-film antireflection coating is not suitable for reducing the Fresnel reflection loss of a homogeneous and anisotropy film.

In our nano-fin, the Goos–Hanchen reflection phase from the structure is not only equal to π but also has phase anisotropy. Therefore, polarization states of forward and backward propagation light are not completely orthogonal in our discussed PB-phase nano-fin system. We thus can realize antireflection in the PB-phase system.

**Figure 3:**
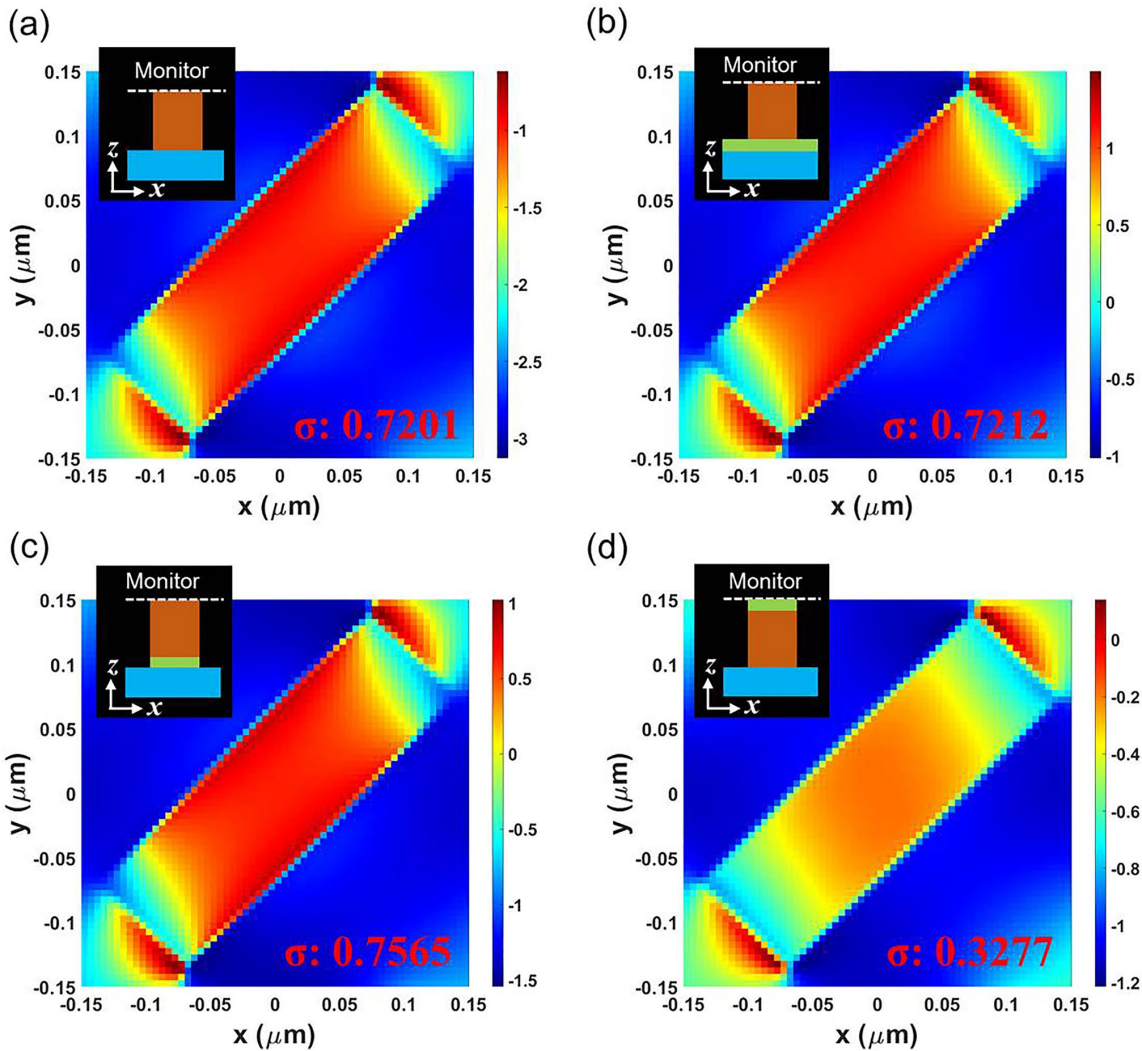
Electric field distribution of nano-fin structures. Simulations of phase distribution at the upper interface of the nano-fin for the (**a**) nano-fin without MgF_2_, (**b**) flat MgF_2_ film, (**c**) nano-fin stacking on the MgF_2_ nano-structure, and (**d**) MgF_2_ nano-structure stacking on a nano-fin. σ is the standard deviation of phase distribution at the upper interface.

Figure 3 presents the phase distribution at the upper interface of the nano-fin coated with four antireflection structures: bare nano-fin, nano-fin with a flat MgF_2_ film, GaN/MgF_2_ nano-fin, and MgF_2_/GaN nano-fin. The corresponding phase distributions are presented in Fig. 3a–d. Here, the thickness of GaN and MgF_2_ was fixed at 950 and 140 nm, respectively. To analyze phase distribution intuitively, we calculated the standard deviation of the phase and marked it as σ. Figure 3a depicts that the σ of the bare nano-fin is 0.7201, which can be considered as a reference. We first observed that the σ of the nano-fin with the flat MgF_2_ film was 0.7212, as shown in Fig. 3b. Compared with the reference, the flat MgF_2_ film contributed inconspicuously to the antireflection effect. The σ of the GaN/MgF_2_ nano-fin was 0.7565. Compared with the flat MgF_2_ film, phase distribution on the interface changed drastically, corresponding to the decreases in efficiency. As shown in Fig. 2d, the GaN/MgF_2_ nano-fin exhibited lower overall PCE at a MgF_2_ thickness of 140 nm. Finally, the σ of the MgF_2_/GaN nano-fin was 0.3277, as shown in Fig. 3d. Compared with the flat MgF_2_ film, the variation in phase distribution on the interface was mitigated and transmittance was increased.

As mentioned above, GaN suffers from absorption loss in the visible range. The optical characteristics of material loss make improving the overall PCE difficult. Therefore, materials without absorption loss in the visible range must be identified. For example, Choudhury et al. comprehensively surveyed a dielectric material for a dielectric metasurface for visible and IR spectral ranges^43^. They suggested silicon nitride (Si_3_N_4_) and titanium oxide (TiO_2_) as good metasurfaces in visible range applications.

**Figure 4:**
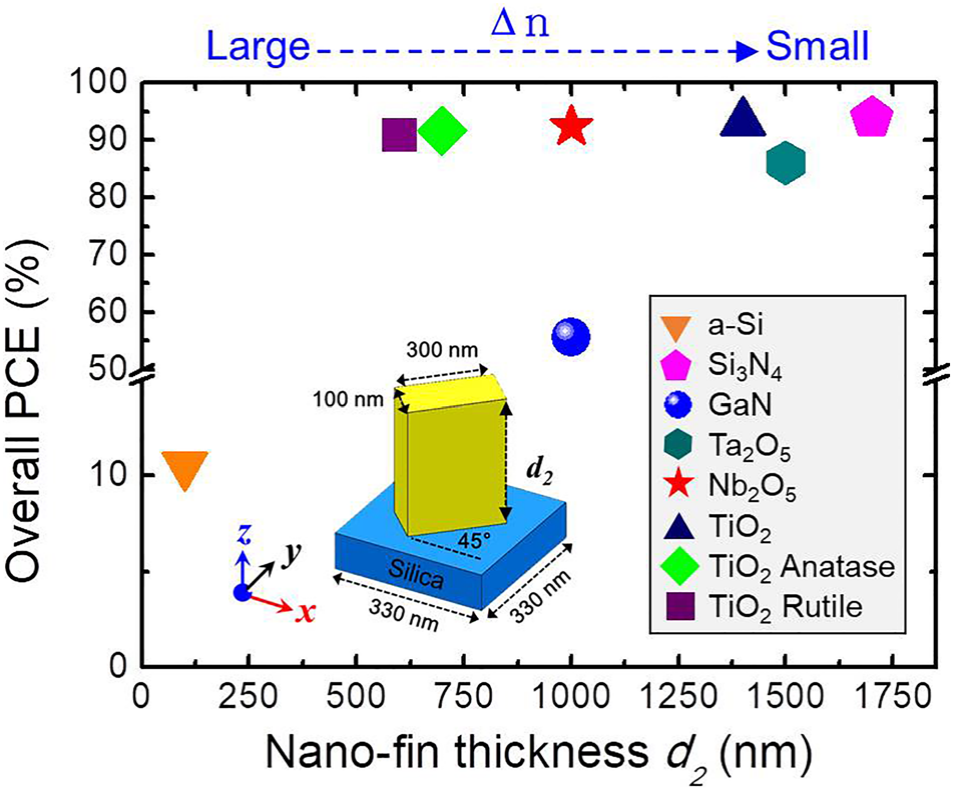
Overall PCE comparison. Optimized thickness (*d*_*2*_) of a nano-fin for various materials at the highest overall PCE. The geometric diagram of a meta-atom is shown in inset.

In addition to absorption loss, the refractive index is crucial for fabrication. Both the propagation phase and anisotropy are positively related to the refractive index. Therefore, a high aspect ratio is required to accumulate sufficient phase modulation for materials with a relatively low refractive index. We thus focused on dielectric materials that can be applied in the visible range and have a high refractive index. Figure 4 depicts the optimized thickness (*d*_*2*_) of nano-fins based on various materials. As shown in the inset of Fig. 4, geometric parameters were all fixed (length, 300 nm; width, 100 nm; and period, 330 nm). Recently, Prof. Tsai’s group demonstrated a GaN structure with an aspect ratio as high as 10–20 for high efficiency^18,44,45^. However, fabricating a nano-fin with such a high aspect ratio is extremely challenging. Thus, we used the geometric parameters of the GaN nano-fin as a benchmark for the state-of-the-art fabrication. Nano-fins with an optimized thickness higher than that of GaN nano-fin were excluded as candidates for a high-efficiency dielectric metasurface. According to this criterion, Si_3_N_4_, tantalum pentoxide (Ta_2_O_5_), and sputtering TiO_2_ are not suitable materials. Compared with GaN, both anatase and rutile TiO_2_ have the advantages of higher efficiency and lower aspect ratio. However, the crystalline phase control of TiO_2_ during deposition is severe. Thus, crystalline TiO_2_ is also excluded. Amorphous silicon (a-Si) is a material with a high refractive index and can be easily processed using standard semiconductor-compatible manufacturing techniques. However, it suffers from huge absorption loss in the visible range, which makes it inappropriate. Finally, Nb_2_O_5_ offers a fair refractive index and low absorption (n + ik = 2.32 + 0i), making it suitable for visible applications. Therefore, we believe that Nb_2_O_5_ is suitable for high-efficient dielectric metasurfaces.

**Figure 5:**
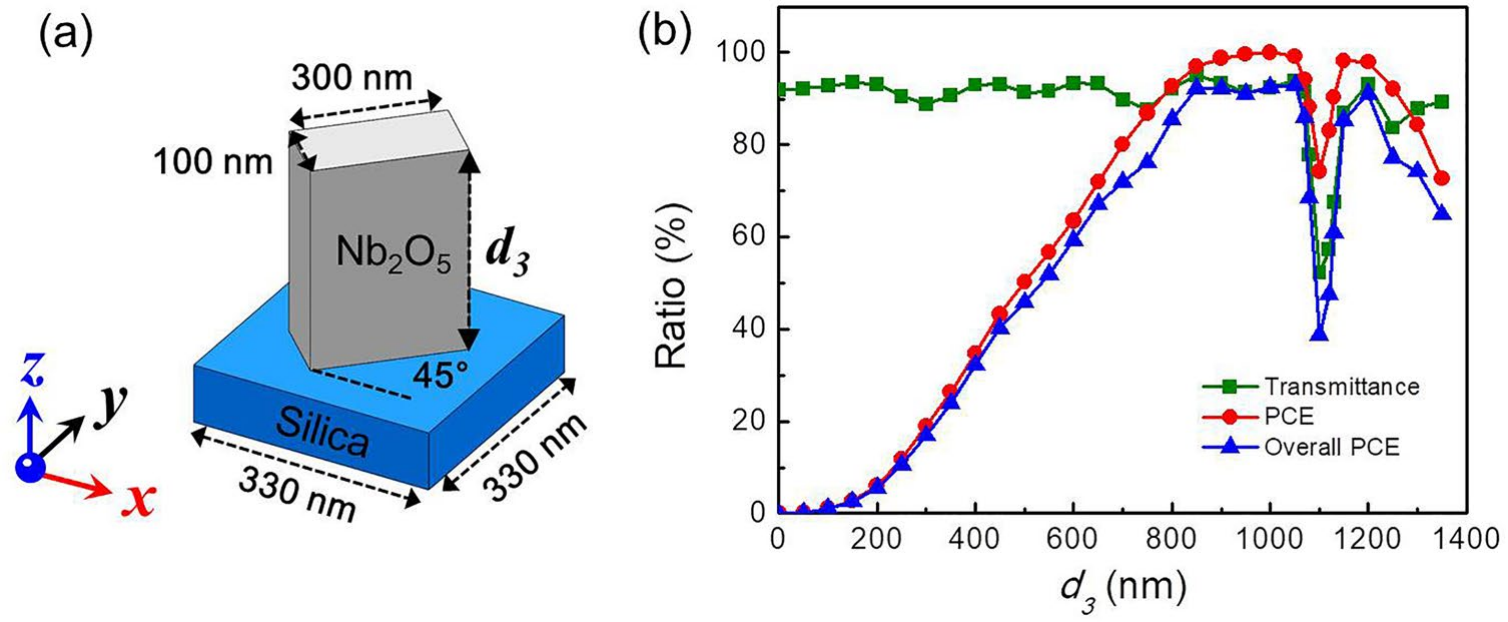
Geometric diagram and optical property of Nb_2_O_5_ nano-fin. (**a**) Schematic of a Nb_2_O_5_ nano-fin on a silica substrate, where *d*_*3*_ is the thickness of the Nb_2_O_5_ nano-fin. (**b**) Transmittance, PCE, and overall PCE versus the thickness of the Nb_2_O_5_ nano-fin. x-polarized light with *λ* = 633 nm impinges perpendicularly onto the structure from bottom along the positive z-direction.

We simulated the optical response of a nano-fin composed of Nb_2_O_5_ patterned on the same substrate at an incident wavelength of 633 nm, as shown in Fig. 5a. The polarization state was XLP propagating along the z-direction. Figure 5b depicts the transmittance, PCE, and overall PCE as a function of the thickness (*d*_*3*_) of the Nb_2_O_5_ nano-fin. Corresponding to the half-waveplate condition for polarization conversion, the peak of the PCE appeared at *d*_*3*_ = 1000 nm. Because Nb_2_O_5_ does not suffer absorption loss in the visible range, which can be easily observed, transmittance did not decrease with an increase in *d*_*3*_. Therefore, same as the PCE, the highest overall PCE appeared at *d*_*3*_ = 1000 nm. As expected, the overall PCE of Nb_2_O_5_ was considerably higher than that of the GaN nano-fin. By contrast, an obvious dip appeared at *d*_*3*_ = 1100 nm, which is caused by guided-mode resonance^46^.

**Figure 6:**
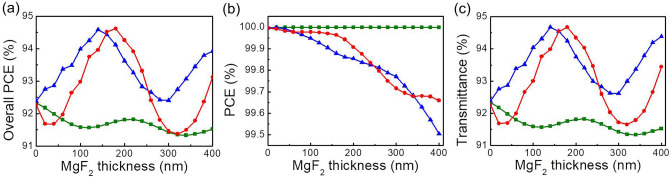
Optical property of Nb_2_O_5_ nano-fin consisted with MgF_2_. Simulation results of (**a**) overall PCE, (**b**) PCE, and (**c**) transmittance as a function of MgF_2_ thickness. The thickness of Nb_2_O_5_ is fixed to be *d*_*3*_ = 1000 nm. The green square denotes a flat MgF_2_ film deposited under the Nb_2_O_5_ nano-fin, the red circle denotes a nano-fin located on the top of the MgF_2_ nano-structure, and the blue triangle denotes a MgF_2_ nano-structure stacked on the top of a nano-fin.

We also considered three different cases of the MgF_2_-based antireflection layer for reducing the reflection loss of the Nb_2_O_5_ nano-fin: a flat MgF_2_ film under the nano-fin, Nb_2_O_5_/MgF_2_ nano-fin, and MgF_2_/Nb_2_O_5_ nano-fin, which are denoted by a green square, red circle, and blue triangle, respectively, in Fig. 6. Here, the thickness (*d*_*3*_) of Nb_2_O_5_ for all cases was 1000 nm. The simulation results of the overall PCE, PCE, and transmittance as a function of MgF_2_ thickness are presented in Fig. 6a–c, respectively. First, a flat MgF_2_ film in the Nb_2_O_5_ system barely contributed to the PCE, overall PCE, and transmittance. Therefore, this case was marked as a reference. Both Nb_2_O_5_/MgF_2_ and MgF_2_/Nb_2_O_5_ nano-fins exhibited obvious enhancement of the overall PCE (Fig. 6a). The highest overall PCE of both Nb_2_O_5_/MgF_2_ and MgF_2_/Nb_2_O_5_ nano-fins was 94.6% when MgF_2_ thickness was 160 and 140 nm, respectively. The thickness of the Nb_2_O_5_ nano-fin (*d*_*3*_ = 1000 nm) was already an optimal optimized half-waveplate. For both Nb_2_O_5_/MgF_2_ and MgF_2_/Nb_2_O_5_ nano-fins, an additional MgF_2_ layer forced the nano-fin away from the optimal optimized thickness of the half-waveplate and decreased the PCE (Fig. 6b). Thus, transmittance enhancement contributed to all the improvement in the overall PCE. Figure 6c shows a transmittance of 94.7% for both Nb_2_O_5_/MgF_2_ and MgF_2_/Nb_2_O_5_ nano-fins. Although both these nano-fins exhibited a good enhancement of the overall PCE, the MgF_2_/Nb_2_O_5_ nano-fin attained thickness efficiency at a lower MgF_2_ thickness. Thus, the antireflection layer located on the top of a nano-fin is considered more suitable.

**Figure 7:**
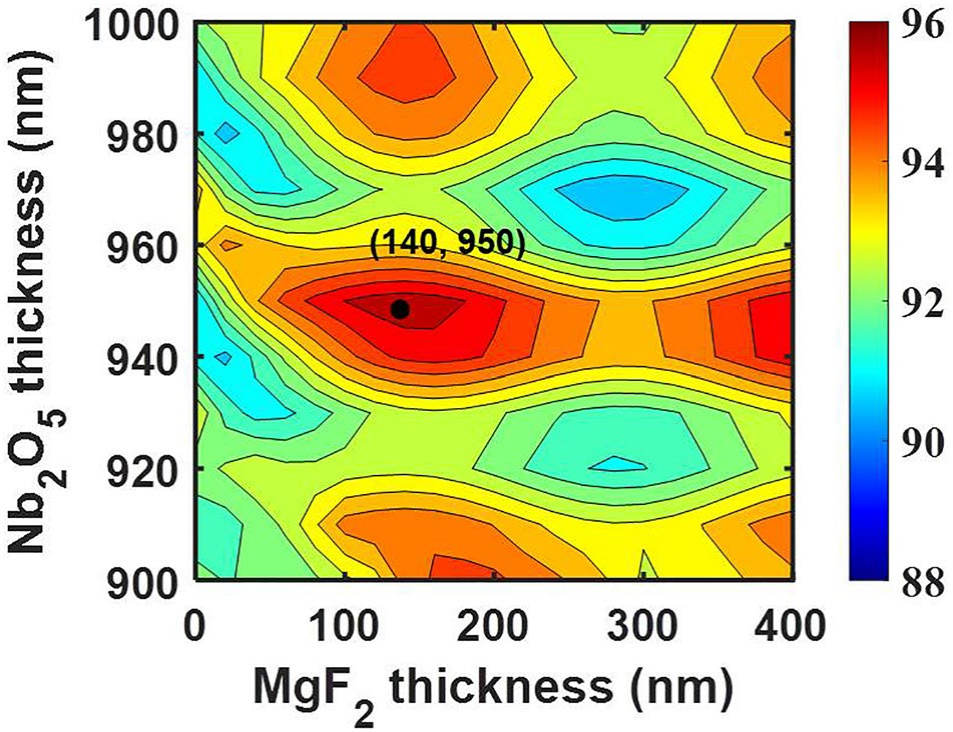
Overall PCE mapping of MgF_2_/Nb_2_O_5_ hetero-nano-fin. Simulation of overall PCE as a function of MgF_2_ and Nb_2_O_5_ thicknesses. MgF_2_ is located on the top of the Nb_2_O_5_ nano-fin. The black dot represents the thickness with the highest overall PCE.

As mentioned above, MgF_2_ contributed negatively to the PCE and reduced the overall PCE when the nano-fin thickness was under an ideal half-waveplate condition. Therefore, we further optimized the hetero-nano-fin to ensure that the Nb_2_O_5_ nano-fin was slightly thinner than the ideal half-waveplate condition. Figure 7 presents the overall PCE as a function of MgF_2_ and Nb_2_O_5_ thicknesses. The color represents the overall PCE. Warm and cold colors represent the enhanced and suppressed overall PCE, respectively, compared with the bare Nb_2_O_5_ nano-fin. In this calculation, MgF_2_ was located on the top of the Nb_2_O_5_ nano-fin. As the thickness of Nb_2_O_5_ was 950 nm, the overall PCE improved from 91 to 96% when MgF_2_ thickness increased from 0 to 140 nm. At this time, MgF_2_ made up for a shortage in the ideal half-waveplate condition. MgF_2_ plays a role in both polarization conversion and antireflection. Moreover, the overall PCE of the MgF_2_/Nb_2_O_5_ nano-fin was 1.7 times higher than that of the MgF_2_/GaN nano-fin. Therefore, we believe that the MgF_2_/Nb_2_O_5_ hetero-nano-fin is a highly efficient candidate for dielectric metasurfaces.

**Figure 8:**
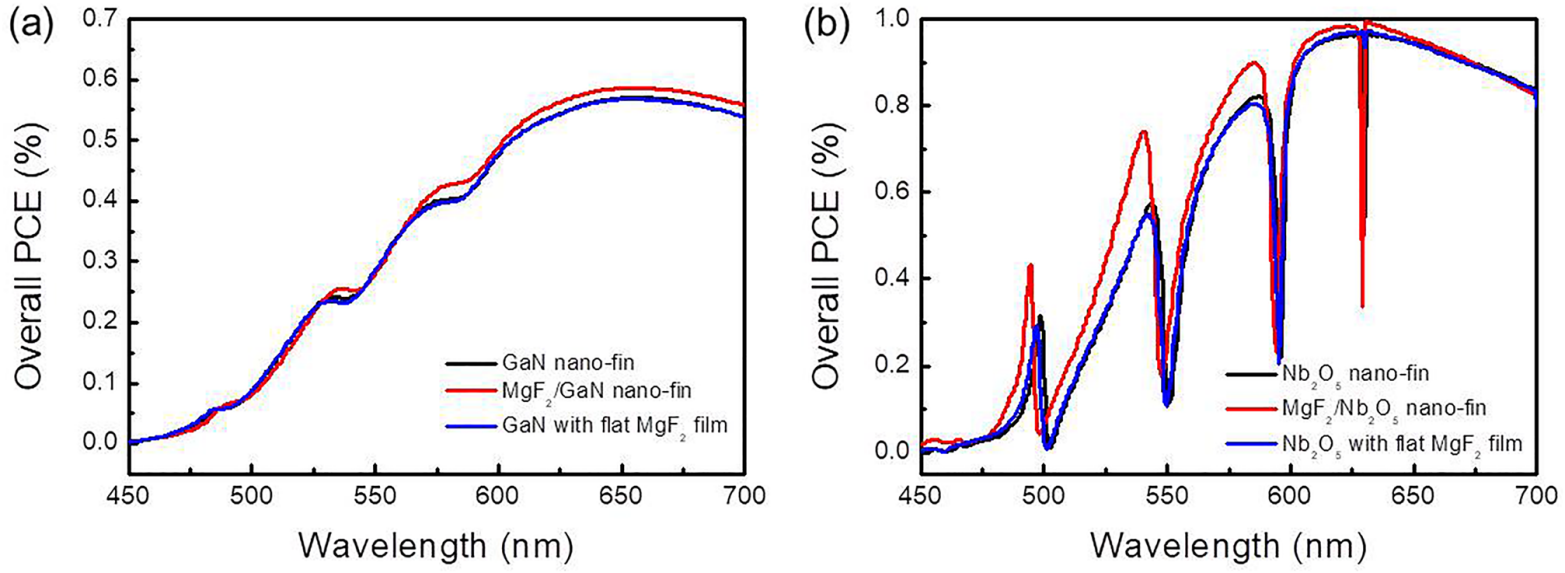
Simulation results of overall PCE spectra. (**a**) GaN nano-fin and flat MgF_2_ film thickness are 950 nm and 140 nm, respectively. In the hetero-nano-fin case, GaN and MgF_2_ thickness is 950 nm and 140 nm. (**b**) For the case of flat MgF_2_ film, the thickness of Nb_2_O_5_ nano-fin and flat MgF_2_ film is 1000 nm and 140 nm, respectively. In the hetero-nano-fin case, Nb_2_O_5_ nano-fin and flat MgF_2_ film thickness is 950 nm and 140 nm, corresponding to the highest overall PCE condition.

We further investigated the overall PCE enhancement of hetero-nano-fin in the visible wave band. Figure 8a depicts the overall PCE of MgF_2_/GaN hetero-nano-fin under normal illumination from 450 to 700 nm. Here, the thickness of GaN and MgF_2_ is 950 nm and 140 nm, corresponding to the highest overall PCE condition. We observed that the antireflection nano-fin worked from 530 to 545 nm and 560 nm to 700 nm. However, the hetero-nano-fin was optimized for 633 nm application. Therefore, the overall PCE enhancement of MgF_2_/GaN nano-fin in the red band is more evident than the blue and green bands. We next investigated the overall PCE enhancement of MgF_2_/Nb_2_O_5_ hetero-nano-fin. Here, the thickness of MgF_2_ and Nb_2_O_5_ is 140 nm and 950 nm, respectively. In the case of flat MgF_2_ (marked as reference), the thickness of MgF_2_ and Nb_2_O_5_ is 140 nm and 1000 nm, corresponding to the highest overall PCE condition. Figure 8b presents that the additional antireflection structure significantly improves efficiency. Compared with the reference, hetero-nano-fin's average overall PCE enhancement is 4.3% from 450 to 700 nm. By contrast, four noticeable dips appear which were caused by guided-mode resonance.

## Conclusions

In summary, we numerically investigated the enhancement of overall PCE of hetero-nano-fins by using GaN and Nb_2_O_5_ nano-fins with three types of antireflection structure: an intuitive flat MgF_2_ layer under nano-fins, nano-fins/MgF_2_, and MgF_2_/nano-fins. Compared with the flat antireflection layer, a hetero-nano-fin exhibited a better overall PCE enhancement. Both GaN/MgF_2_ and MgF_2_/GaN nano-fins improved PCE with an increase in MgF_2_ thickness. Compared with the GaN/MgF_2_ nano-fin, the MgF_2_/GaN nano-fin led to higher overall PCE enhancement. For the MgF_2_/GaN nano-fin, the overall PCE improved from 52.7% to 54% with an increase in MgF_2_ thickness from 0 to 140 nm. Unfortunately, the absorption loss of GaN in the visible range limited its overall PCE. By contrast, lossless Nb_2_O_5_ was considered because of its high refractive index. The overall PCE of the Nb_2_O_5_-based nano-fin was 1.7 times higher than that of the GaN-based nano-fin. Moreover, an overall PCE up to 96% was achieved after optimization. The overall PCE spectra show that Nb_2_O_5_-based hetero-nano-fin's average overall PCE enhancement is 4.3% from 450 to 700 nm. Antireflection coating for a metasurface is crucial for enhancing the efficiency and reducing the background signal of a meta-device.

## Methods

All simulation results were performed with the FDTD methods. All nano-fins were simulated with periodic boundary conditions in xz and yz-planes; absorbing boundary in xy-planes. The incident wavelength was 633 nm with x-polarized. The refractive index of silica substrate, GaN, and Nb_2_O_5_ are 1.45 + 0i, 2.29 + 0.061i, and 2.32 + 0i at 633 nm, respectively. The refractive index of Nb_2_O_5_ is measured by spectroscopic ellipsometry. The 3D model of an infinite array of dielectric nano-fins implemented periodic boundary conditions (PBC) on the sides and perfectly matched layers (PML) on the top and the bottom of the simulation domain. The mesh size was set to be smaller than 5 nm. The naon-fins were excited by a normal-incidence plane wave from the substrate-side.

## Data Availability

All data generated or analysed during this study are included in this published article (and its Supplementary Information files).
